# Black Americans’ Diminished Return of Educational Attainment on Tobacco Use in Baltimore City

**DOI:** 10.1007/s40615-023-01805-0

**Published:** 2023-09-27

**Authors:** Rifath Ara Alam Barsha, Shervin Assari, Mian B. Hossain, Jummai Apata, Payam Sheikhattari

**Affiliations:** 1 Center for Urban Health Disparities Research and Innovation, Morgan State University, Baltimore, MD USA; 2 Department of Family Medicine, Charles R Drew University of Medicine and Science, Los Angeles, CA USA; 3 Department of Urban Public Health, Charles R Drew University of Medicine and Science, Los Angeles, CA USA; 4 School of Community Health and Policy, Morgan State University, Baltimore, MD USA; 5 The Prevention Sciences Research Center, School of Community Health and Policy, Morgan State University, Baltimore, MD USA

**Keywords:** Smoking, Flavored tobacco, Social determinants, Educational attainment, Marginalized groups

## Abstract

**Background:**

Socioeconomic status (SES) indicators such as educational attainment are fundamental factors affecting health. One mechanism through which education affects health is by reducing the likelihood of engaging in high-risk behaviors such as smoking. However, according to the marginalization-related diminished returns (MDRs) theory, the association between education and health may be weaker for marginalized populations such as Black than White, primarily due to racism and discrimination. However, little is known about the racial variations in the differential associations between educational attainment and tobacco use in a local setting.

**Aim:**

This study aimed to investigate the differential association between educational attainment and tobacco use among racial groups in a community sample in Baltimore City.

**Methods:**

This cross-sectional study used data from a community survey conducted in 2012–2013 in Baltimore City among adults aged 18 years or older. The participants were 3501 adults. Univariate, bivariate, and logistic regression analyses were performed using Stata to investigate the racial difference in the association between education and two outcomes: current smoking status and menthol tobacco product use.

**Results:**

The study found that adults with a graduate degree were less likely to be current smokers (adjusted odds ratio [AOR]: 0.10, 95% confidence interval [CI]: 0.08–0.13) and menthol tobacco users (AOR: 0.10, 95% CI: 0.07–0.14) compared to those with less than high school diploma. The inverse associations between educational attainment and current smoking (AOR: 1.83, 95% CI: 1.05–3.21) and menthol tobacco product use (AOR: 4.73, 95% CI: 2.07–10.80) were weaker for Back individuals than those who were White.

**Conclusion:**

Due to MDRs of educational attainment, while highly educated White adults show a low risk of tobacco use, educated Black adults remain at a disproportionately increased risk. The study emphasizes the need for better policies and programs that address minorities’ diminished return of education for tobacco use.

## Introduction

Despite recent declines in smoking prevalence over the past decade, tobacco remains the leading preventable cause of morbidity and death in the United States (US) [18, 19]. Approximately 30 million US adults aged 18 years or older still smoke, and smoking causes 480,000 deaths (about 1 in 5 deaths) each year [18, 19]. The burden of smoking-related chronic diseases is also huge: more than 16 million US adults have such health conditions [18, 19]. Smoking also remains the foremost cause of mortality and morbidity associated with lung cancer and cardiovascular disease [56]. When considering direct and indirect expenses, smoking imposed a burden of more than 600 billion in 2018 on the US healthcare system [18, 19].

The impact of tobacco usage is not evenly distributed across society, as individuals with higher socioeconomic status (SES) are less likely to use tobacco and experience a lower risk of tobacco-related diseases [18, 19, 34, 37]. In addition, educational attainment, a key indicator of SES, has a major effect on smoking behaviors, where with increasing education levels smoking rates generally decrease [2, 31, 34]. Racial/ethnic minorities experience wide disparities in smoking behaviors and tobacco-related diseases. The prevalence of smoking is lower among Black youth; however, this advantage does not result in low prevalence of smoking when transition to adulthood [1]. Despite having similar smoking prevalence among Black and White adults, Black adults are less likely to quit [26] and experience a larger burden of tobacco-related diseases — a well-known Black smoking paradox among tobacco researchers. Additionally, the use of mentholated cigarettes is historically higher among Black smokers [30, 32, 62].

However, marginalization-related diminished returns (MDRs) theory [6] and associated empirical evidence [7] indicate that the effect of SES and race/ethnicity on tobacco use is not independent. Instead, the effect of SES on tobacco use widely varies among racial/ethnic groups [7], and there are racial/ethnic disparities across all SES categories. The protective effects of SES indicators, such as educational attainment, on tobacco use appear to be less pronounced for Black population compared to White population. This finding is observed in national studies examining use of e-cigarette [12], hookah [13], and traditional cigarette [11] among Black and Latino youth and adults. However, no research has been conducted in Baltimore City utilizing local data to investigate the diminished gains of SES resources among marginalized groups in the field of tobacco use.

Therefore, this study aims to investigate the association between educational attainment and tobacco use among racial and ethnic groups in a community sample in Baltimore City. This current study will be the first to examine the differential returns of SES among racial groups in Baltimore City. Considering the city’s well-known history of segregation, inequalities, and place-based practices [25], it is important to investigate the unequal effect of SES on tobacco use among different racial groups. We hypothesized that a weaker protective effect of educational attainment on tobacco use (current smoking and menthol tobacco product use) will be observed for Black adults than for White adults.

## Methods

### Study Setting and Sample

The study used data from a community survey conducted in Baltimore City in 2012–2013 by CEASE (communities engaged and advocating for a smoke-free environment) initiative of the Morgan State University (MSU). CEASE is a community-based participatory research (CBPR) initiative between MSU researchers and Baltimore City’s underserved communities and aims to reduce tobacco-related health disparities through interventions and cessation programs [59]. As a part of a project, a self-administered and paper-based community survey was conducted among adults aged 18 years and older, regardless of their smoking status, to ascertain the community’s needs concerning tobacco exposure and smoking habits. Participants were recruited by trained former smokers called peer motivators at certain community events (i.e., farmers’ market, church services, health fairs) in Baltimore City. The peer motivators approached and asked individuals at the event to take a brief community survey on tobacco use. A total of 3931 adults who consented, participated in the survey.

### Analytical Sample

In the data analysis of this current study, only Black and White adults were included because the number of respondents of other races (Asians, Native Americans, Latinos, and others) was too small to make any meaningful comparisons. Additionally, participants with missing data for the variables used in his study were also excluded. Sixteen participants did not provide information about their race and were excluded. All the participants responded to the question used to define one of the dependent variables (current smoking status) of the study. A total of 52 participants (Black, *n* = 49 and White, *n* = 3) did not respond to the question that captured menthol tobacco product use and were excluded from the study. After excluding those, the final sample for the analysis of this study was 3501.

### Ethical Consideration

The Institutional Review Board (IRB) of Morgan State University granted approval for the research project. To ensure confidentiality and minimize the risk of a data breach, no personal identifiers were used during the data analysis process. All participants were adults and provided written consent.

### Measures

#### Outcome Variables

This study has two outcome variables: current smoking status and menthol tobacco products use. Current smoking status was measured using the following item: “Do you currently smoke?” Participants were presented with response options limited to “yes” or “no”. Those who indicated “yes” were classified as current smokers. This item has been used by others [51, 57], however, smoking literature shows inconsistency in items used to measure smoking status [44, 60]. Many prominent national behavioral surveillance systems, including the National Health and Nutrition Examination Survey (NHANES), have utilized self-reported measure of smoking status [17]. In addition, self-reported smoking has shown strong reliability and validity [49, 61]. Menthol tobacco product use was measured by the following questions: “What type of tobacco products do you use?” The response options were regular, light, or menthol, and those who responded “menthol” were considered menthol tobacco product users. “Regular” cigarettes and tobacco products were found to be frequently used in the previous research to distinguish from flavored tobacco products [21, 29]. “Light” cigarettes were redesigned forms of cigarettes with certain features that were marketed with such labeling [42]. The question was asked only to those who reported being current smokers. Menthol tobacco has been linked to increased nicotine dependence and decreased cessation [29, 58], making it an important outcome to investigate.

#### Independent Variable

Education is the independent variable in this study. The educational level was reported as (1) Some high school or less, (2) graduated from high school, (3) one or more years of college, (4) graduated from trade school, and (5) graduated from college.

#### Demographic Covariates

Age and gender were included as covariates when adjusting for confounding. Age was operationalized as a categorical variable (18–29 years, 30–39 years, 40–49 years, 60 years and more), and gender was a dichotomous variable (female = 0 and male = 1).

#### Moderator

Race is the moderating variable and self-identified. Only Black and White adults were included in the data analysis as the aim is to test Blacks–White differences.

### Statistical Analysis

Data analysis was performed using Stata 15.0 (Stata Corp, College Station, TX: StataCorp LLC). The univariate analysis results were presented as frequencies and percentages. For bivariate analysis, Pearson chi square tests were used to compare Black and White participants. Four sets of binary logistic regression models were estimated for multivariable analysis. First, two logistic regressions were performed in the pooled sample. Model 1 did not include educational attainment by race interaction terms. The race-by-educational attainment interaction terms were estimated in model 2. Then, race-specific logistic regressions were performed (model 3 for Black adults and model 4 for White adults). The stratified models were estimated to understand if the effects of covariates are similar across groups. However, the inference regarding presence of diminished returns was based on model 2. The logistic regression results are presented as adjusted odds ratios (ORs) and 95% confidence intervals (CIs). Significance levels were set at *P* ≤ 0.05, and significant *P* values were also reported.

## Results

Table 1 provides descriptive statistics of the study variables in the overall sample and by race. This study included 3501 adults who were either Black (*n* = 2428, 69%) or White (*n* = 1073, 31%) adults. Only 14% of Black individuals were college graduates compared to 47% of White individuals. About 53% of Black adults reported being a current smoker versus 32% of White adults. Menthol tobacco product use was also more common among Black than White adults (39.1% vs. 17.2%). In comparison, Baltimore City had 62.8% Black and 30.3% White population in 2017 [14] which was closer to the time of this study. Attainment of a bachelor’s degree or higher in individuals 25 years or older in Baltimore City was 19% in Blacks and 60% in Whites [15]. The prevalence of tobacco use was 31.3% in Blacks and 18.5% in Whites in Baltimore City [41].

**Table 1:** Descriptive statistics in the overall sample and by race

Variables	All (*n* = 3501)	Black (*n* = 2428)	White (*n* = 1073)
	*N* (%)	*N* (%)	*N* (%)
Education^***^			
Some high school or less	773 (22.1)	616 (25.4)	157 (14.6)
Graduated from high school	1164 (33.3)	917 (37.8)	247 (23.0)
One or more years of college	521 (14.9)	392 (16.1)	129 (12.0)
Graduated from trade school	190 (5.4)	155 (6.4)	35 (3.3)
Graduated from college	853 (24.3)	348 (14.3)	505 (47.1)
Age (years)^***^			
18–29	964 (27.5)	577 (23.8)	387 (36.1)
30–39	633 (18.1)	420 (17.3)	213 (19.8)
40–49	766 (21.9)	595 (24.5)	171 (15.9)
50–59	723 (20.7)	559 (23.0)	164 (15.3)
60 and more	415 (11.8)	277 (11.4)	138 (12.9)
Gender^*^			
Female	1783 (50.9)	1208 (49.7)	575 (53.6)
Male	1718 (49.1)	1220 (50.3)	498 (46.4)
Current smoker^***^			
Yes	1624 (46.4)	1274 (52.5)	350 (32.6)
No	1877 (53.6)	1154 (47.5)	723 (67.4)
Menthol tobacco product use^***^			
Yes	1134 (32.4)	949 (39.1)	185 (17.2)
No	2367 (67.6)	1479 (60.9)	888 (82.8)

^*^p < 0.05; **p < 0.01; *** *p* < 0.001 for comparison of Black and White adults

Table 2 describes the prevalence of current smoking and menthol tobacco product use across race and educational attainment intersectional groups. The current smoking among Black individuals with lower education (less than high school) was 69.5%. The prevalence was similar for low-educated (less than high school) White individuals (68.9%). The prevalence was lowest among highly educated (college graduates) Black individuals (19.5%) and White individuals (11.1%). Menthol tobacco product use among low-educated (less than high school) Black individuals was 50.7%. Those who were Whites and had graduated from trade school had the highest percentage of exposure (97.9%).

**Table 2:** Prevalence of current smoking and menthol tobacco product use across race and educational attainment intersectional groups

Education	Current smoking		Menthol tobacco Product use	
	Black *n* (%)	White *n* (%)	Black *n* (%)	White *n* (%)
Some high school or less	428 (69.5)	108 (68.9)	312 (50.7)	62 (39.5)
Graduated from high school	545 (59.4)	118 (47.8)	413 (45.0)	73 (29.6)
One or more years of college	152 (38.8)	48 (37.2)	118 (30.1)	26 (20.2)
Graduated from trade school	81 (52.3)	20 (57.1)	63 (40.7)	15 (42.7)
Graduated from college	68 (19.5)	56 (11.1)	43 (12.4)	9 (1.78)

Table 3 presents the results of four logistic regression models with educational attainment as the independent variable and current smoking status as the dependent variable. While model 1 only included the main effects of education and race, model 2 also included an interaction term between race and education. Model 3 was estimated among Black adults and model 4 among White adults. Based on model 1, high education was associated with lower odds of current smoking. College graduates were significantly less likely to be current smokers than those who attended some high school or less (AOR: 0.10, 95% CI: 0.08–0.13). A significant interaction between race and education on current smoking was observed in model 2, suggesting that the protective effects of education on current smoking were larger for White than Black adults (AOR: 1.83, 95% CI:1.05–3.21). Model 3 provided evidence of significantly lower odds of being a current smoker for college graduates who were Black adults (AOR: 0.12, 95% CI: 0.09–0.17). Model 4 also showed a significant protective effect of education on current smoking for White individuals (AOR: 0.05, 95% CI: 0.03–0.09).

**Table 3:** Logistic regression on education and current smoking

Variables	All (*n* = 3501)		Black (*n* = 2428)	White (*n* = 1073)
	Model 1	Model 2	Model 3	Model 4
	AOR (95% CI)	AOR (95% CI)	AOR (95% CI)	AOR (95% CI)
Education^***^				
Some high school or less	Ref	Ref	Ref	Ref
Graduated from high school	0.59^***^ (0.48–0.73)	0.42^***^ (0.27–0.66)	0.65^***^ (0.52–0.82)	0.41^***^ (0.26–0.63)
One or more years of college	0.32^***^ (0.25–0.41)	0.30^***^ (0.18–0.50)	0.33^***^ (0.25–0.44)	0.26^***^ (0.15–0.44)
Graduated from trade school	0.50^***^ (0.36–0.71)	0.59 (0.26–1.29)	0.49^***^ (0.34–0.73)	0.60 (0.27–1.30)
Graduated from college	0.10^***^ (0.08–0.13)	0.06^***^ (0.04–0.10)	0.12^***^ (0.09–0.17)	0.05^***^ (0.03–0.09)
Age^***^				
18–29	Ref	Ref	Ref	Ref
30–39	1.67^***^ (1.32–2.10)	1.64^***^ (1.30–2.07)	1.73^***^ (1.31–2.28)	1.45 (0.94–2.22)
40–49	2.85^***^ (2.28–3.56)	2.78^***^ (2.26–3.48)	2.80^***^ (2.16–3.63)	2.93^***^ (1.87–4.58)
50–59	2.69^***^ (2.15–3.38)	2.66^***^ (2.12–3.33)	3.30^***^ (2.53–4.31)	1.29 (0.81–2.04)
60 and more	1.08 (0.83–1.40)	1.06 (0.81–1.38)	1.27 (0.93–1.74)	0.59 (0.35–1.00)
Gender (male)	2.22^***^ (1.91–2.59)	2.24^***^ (1.92–2.60)	2.43^***^ (2.03–2.90)	1.66^**^ (1.23–2.25)
Race (Black)	1.28^**^ (1.07–1.53)	0.96 (0.65–1.43)	NA	NA
Race*education				
Some high school or less	NA	Ref	Ref	Ref
Graduated from high school	NA	1.52 (0.93–2.49)	NA	NA
One or more years of college	NA	1.09 (0.61–1.94)	NA	NA
Graduated from trade school	NA	0.84 (0.35–2.01)	NA	NA
Graduated from college	NA	1.83^*^ (1.05–3.21)	NA	NA

Significance *p < 0.05; **p < 0.01; *** *p* < 0.001, *AOR* adjusted odds ratio, *CI* confidence interval

Table 4 presents the results of four logistic regression models with education as the independent variable and the use of menthol tobacco products as the dependent variable. Based on model 1, there was a significant association between higher education and the use of menthol tobacco products. College graduates were significantly less likely to use menthol tobacco products than those who attended some high school or less (AOR: 0.10, 95% CI: 0.07–0.14). A significant interaction between race and education on menthol tobacco product use was observed in model 2, suggesting that the protective effects of education on menthol tobacco products are larger for White adults than Black adults (AOR: 4.73, 95% CI: 2.07–10.80). Model 3 showed that higher education was significantly associated with lower odds of using menthol tobacco products for Black adults (AOR: 0.17, 95% CI: 0.12–0.25). Model 4 also showed a significant protective effect of education on menthol tobacco product use for White adults (AOR: 0.02, 95% CI: 0.01–0.04).

**Table 4:** Logistic regression on education and menthol tobacco product use

Variables	All (*n* = 3501)		Black (*n* = 2428)	White (*n* = 1073)
	Model 1	Model 2	Model 3	Model 4
	AOR (95% CI)	AOR (95% CI)	AOR (95% CI)	AOR (95% CI)
Education^***^				
Some high school or less	Ref	Ref	Ref	Ref
Graduated from high school	0.79^*^ (0.65–0.95)	0.68 (0.44–1.05)	0.83 (0.67–1.03)	0.58^*^ (0.37–0.91)
One or more years of college	0.49^***^ (0.38–0.63)	0.45^**^ (0.25–0.78)	0.52^***^ (0.39–0.69)	0.33^***^ (0.19–0.58)
Graduated from trade school	0.76 (0.54–1.07)	1.18 (0.54–2.55)	0.71 (0.49–1.04)	1.20 (0.55–2.62)
Graduated from college	0.10^***^ (0.07–0.14)	0.03^***^ (0.01–0.07)	0.17^***^ (0.12–0.25)	0.02^***^ (0.01–.04)
Age^***^				
18–29	Ref	Ref	Ref	Ref
30–39	1.69^***^ (1.31–2.18)	1.65^***^ (1.28–2.13)	1.70^***^ (1.26–2.29)	1.40 (0.84–2.32)
40–49	2.35^***^ (1.86–2.97)	2.29^***^ (1.81–2.89)	2.79^***^ (2.14–3.63)	1.09 (0.65–1.83)
50–59	2.15^***^ (1.70–2.72)	2.09^***^ (1.65–2.66)	2.91^***^ (2.23–3.81)	0.47^*^ (0.26–0.84)
60 and more	0.97 (0.72–1.31)	0.95 (0.70–1.27)	1.30 (0.93–1.82)	0.21^***^ (0.10–0.44)
Gender (male)	2.09^***^ (1.78–2.46)	2.10^***^ (1.79–2.47)	2.36^***^ (1.97–2.83)	1.11 (0.77–1.61)
Race (Black)	1.90^***^ (1.56–2.31)	1.54^*^ (1.06–2.23)	NA	NA
Race*education				
Some high school or less	NA	Ref	NA	NA
Graduated from high school	NA	1.19 (0.74–1.94)	NA	NA
One or more years of college	NA	1.12 (0.60–2.07)	NA	NA
Graduated from trade school	NA	0.59 (0.25–1.39)	NA	NA
Graduated from college	NA	4.73^***^ (2.07–10.80)	NA	NA

Significance *p < 0.05; **p < 0.01; *** *p* < 0.001, *AOR* adjusted odds ratio, *CI* confidence interval

## Discussion

This study has two main findings. First, in the pooled sample that included Black and White adults in Baltimore city, higher educational attainment, such as college degree, was associated with lower odds of smoking and use of menthol tobacco products compared to less than high school diploma. Second, higher education had a smaller protective association with current smoking and menthol tobacco product use for Black adults than for White adults.

The current study’s finding on the inverse association between educational attainment and tobacco use is supported by previous research on SES resources and health outcomes [28, 31, 52]. According to the theories by Link and Phelan [46], Mirowsky and Ross [40], and Marmot [39], higher resources, such as educational attainment, are linked to better health and well-being. A study found that higher education is associated with the perception of more smoker-related stigma that could lead smokers to quit [54].

The observed racial variations in the impact of education on smoking status and menthol tobacco product use align with a pattern where White individuals tend to experience larger protective effects from SES resources compared to Black individuals across multiple other risk factors [4, 6, 8]. The findings on smaller protective effects of educational attainment on tobacco use (current smoking and menthol tobacco products use) among Black adults, in comparison to White adults, are in line with findings from other studies examining the racial differences in the effects of SES on other related behavioral and health outcomes such as self-rated health [8], alcohol consumption [3], physical activity [9], and depression [5].

Given that smoking is one of the significant behavioral risk factors for disparities in morbidity and mortality in the USA in Black populations [18, 19, 43], our findings are of great importance. The study’s results shed light on the disparities in smoking and flavored tobacco product use, such as menthols, revealing that the impact is disproportionately worse for the Black population than their White counterparts. The observed racial disparities in the protective effect of higher education on smoking status and menthol tobacco product use may stem from inequities within the education system [23] and other social institutions. The limited availability of educational resources in predominantly Black neighborhoods could contribute to lower educational quality, thereby accounting for the differential returns of education on smoking prevalence and flavored-tobacco product use across racial groups. In addition, the impact of education on health and behavioral outcomes is influenced by the extent to which education can translate into income and wealth (i.e., the utility of education), a process that is affected by the racialization of Black populations. Despite anti-discrimination regulations in the labor market, Black individuals experience fewer employment opportunities than White individuals [26]. Moreover, highly educated Black adults do not have equal access to opportunities as White adults [33]. These mechanisms collectively could lead to diminished health gains from education for Black people compared to the White population.

The differential returns of educational attainment on reducing the current smoking and menthol tobacco product use for Black adults may be partially attributable to the practices of tobacco industry, as well as disparities in access to high-efficacy smoking cessation services. Evidence is suggestive of the presence of predatory marketing practices in disadvantaged and Black/Latino neighborhoods, with specific flavor branding targeted toward certain racial groups [36, 50]. For example, mentholated cigarettes are targeted to predominantly Black neighborhoods [20, 36]. In their recent research, Choi et al. [24] found an increase in education-related disparities in current smoking among the non-Hispanic Black population. They discussed that it could be related to the use of menthol cigarettes as it increases nicotine dependence and is hard to quit. In addition, Black individuals often experience limited access to smoking cessation programs in urban areas [22]. Even when access to cessation programs is available, the effectiveness of such services is comparatively lower for Black individuals than White individuals [22].

A major contribution of this study was to document diminished returns of education for Black residents of Baltimore. Baltimore has a long history of residential segregation characterized by deep racial and spatial divides [25]. Due to years of discriminatory policies and practices, the hypersegregated Baltimore City neighborhoods encounter profoundly different lived experiences [16]. While the White neighborhoods accumulate structured advantages, those opportunities are scarce in Black neighborhoods [16]. In addition, concentrated investment in Baltimore city is well documented with predominantly Black neighborhoods receiving four times less investments than neighborhoods with fewer Black population [38]. In Baltimore, housing discrimination has caused underfunded schools in Black communities, resulting in low-quality education [45]. In addition, racial discrimination and residential segregation profoundly impact an individual’s health and well-being as it affects health in various ways, such as access to quality education, a health-promoting environment, and access to health care [35]. Substantial racial difference in health outcomes exists in Baltimore City, where those who are Black bear a disproportionate burden of disease. Compared to White individuals, the rate of obesity, diabetes, high blood pressure, smoking prevalence, and childhood asthma is higher for Black individuals in the city [35]. For example, one recent statistic shows that 31.3% of Black adults are current tobacco users compared to 18.5% of White adults [41]. Though national statistics shows similar smoking rate among Black and White adults, Baltimore City Black people have a higher prevalence than Whites. This could be due to the stress associated with upward mobility, racial discrimination, and disadvantages linked with residential segregation such as low-quality education, which may cause Black people to use tobacco and other substances to cope with the stress of discrimination they experience [46]. In addition, the most recent available race-specific statistics showed that the rate of tobacco use did not change consistently among Baltimore City Black adults across the years: 31.2% in 2012 and 31.3% in 2018 [41]. Therefore, even though utilizing data collected in 2012 and 2013, this research holds the promise of offering critical insights into tobacco use among Baltimore City adults. Considering the inequities that exist in Baltimore City and disparities experienced by the Black population in the city, this study provided crucial information regarding unequal gain of SES resources such as education across racial groups. The study findings emphasize the need to shift the focus of policymakers exclusively from low SES populations to include high SES racial minorities also. It also denotes the need to design and adopt different strategies across racial groups to address tobacco-related and other health disparities. In addition, addressing health disparities requires strategies and interventions that goes beyond equalizing socioeconomic resources for Black population.

Although tobacco prevention activities have been successful in reducing overall smoking rates in the USA, there still exist disparities within SES and race strata [43]. Although higher educational attainment has been shown to be associated with lower smoking rates [34, 37], this protective effect of education on smoking was not as prominent in the Black participants in Baltimore. These diminishing returns have been identified for smoking and other health issues [3, 5, 8–10]. This finding is important for guiding smoking cessation policies and interventions by focusing strategies across and within SES and racial strata rather than SES only in order to close the smoking and attendant health disparities that occur across and within SES and racial groups.

The current study had a few limitations. The cross-sectional design of the study does not allow causal inference. We can only infer association rather than causation from these data. In addition, smoking was measured using self-reported data. However, self-reported measure of smoking is widely accepted and used in many behavioral risk factors surveillance systems both in the US and worldwide and is a reliable and valid measure of current smoking status [27, 48, 49, 53]. In addition, the study could not differentiate between the regular and occasional smoker and could possibly underreport the smoking status. Inconsistencies in the measurement and definition of smoking and tobacco use are a remaining challenge in the field of tobacco epidemiology [44, 60]. There were some omitted variables, and generalizability of the sample was also limited. Finally, the study did not use a random sample, and the results are not generalizable to the US population. Convenience sampling however has its advantages such as being cost effective, efficient and easier to implement, particularly for community-based interventions. Despite these limitations, the study results suggest that MDRs observed in national surveys also hold locally in Baltimore. More research is needed on policies that can undo such disparities.

## Conclusion

Although an inverse association between educational attainment and tobacco use was detected in our overall sample of Black adults in Baltimore City, this association was weaker for Black individuals, which can be explained by marginalization-related diminished returns of resources and assets, possibly due to social stratification, segregation, racism, and discrimination. More local research is needed to test how tobacco control policies may alter such disparities. Provision of cessation services in Black communities should go beyond low SES individuals and recruit smokers across the full SES spectrum. For Whites, however, such programs can be more concentrated in low SES sections of the society.

## Data Availability

Data are not public. Data will be available upon request to the authors.
